# The microbiome is dispensable for normal respiratory function and chemoreflexes in mice

**DOI:** 10.3389/fphys.2024.1481394

**Published:** 2024-12-06

**Authors:** Savannah Lusk, Nicoletta K. Memos, Andrea Rauschmayer, Russell S. Ray

**Affiliations:** ^1^ Department of Neuroscience, Baylor College of Medicine, Houston, TX, United States; ^2^ Baylor College of Medicine, McNair Medical Institute, Houston, TX, United States

**Keywords:** microbiome, breathing, dysbiosis, germ-free, mice

## Abstract

Increasing evidence indicates an association between microbiome composition and respiratory homeostasis and disease, particularly disordered breathing, such as obstructive sleep apnea. Previous work showing respiratory disruption is limited by the methodology employed to disrupt, eliminate, or remove the microbiome by antibiotic depletion. Our work utilized germ-free mice born without a microbiome and described respiratory alterations. We used whole-body flow through barometric plethysmography to assay conscious and unrestrained C57BL/6J germ-free (GF, n = 24) and specific-pathogen-free (SPF, n = 28) adult mice (with an intact microbiome) in normoxic (21% O_2_,79% N_2_) conditions and during challenges in hypercapnic (5% CO_2_, 21% O_2_, 74% N_2_) and hypoxic (10% O_2_, 90% N_2_) environments. Following initial plethysmography analysis, we performed fecal transplants to test the ability of gut microbiome establishment to rescue any observed phenotypes. Data were comprehensively analyzed using our newly published respiratory analysis software, *Breathe Easy*, to identify alterations in respiratory parameters, including ventilatory frequency, tidal volume, ventilation, apnea frequency, and sigh frequency. We also considered possible metabolic changes by analyzing oxygen consumption, carbon dioxide production, and ventilatory equivalents of oxygen. We also assayed GF and SPF neonates in an autoresuscitation assay to understand the effects of the microbiome on cardiorespiratory stressors in early development. We found several differences in baseline and recovery cardiorespiratory parameters in the neonates and differences in body weight at both ages studied. However, there was no difference in the overall survival of the neonates, and in contrast to prior studies utilizing gut microbial depletion, we found no consequential respiratory alterations in GF *versus* SPF adult mice at baseline or following fecal transplant in any groups. Interestingly, we did see alterations in oxygen consumption in the GF adult mice, which suggests an altered metabolic demand. Results from this study suggest that microbiome alteration in mice may not play as large a role in respiratory outcomes when a less severe methodology to eliminate the microbiome is utilized.

## 1 Introduction

Respiratory dysregulation is a complex puzzle in medicine, where the interplay of various physiological systems often blurs the line between cause and consequence. One such relationship exists between microbiota imbalance, i.e., dysbiosis, and respiratory control across the nervous system. Previous work suggests that gut dysbiosis may not be merely a symptom. Rather, it could play a contributory or even causative role in the pathogenesis of nervous system disorders that impinge upon respiratory function (de Vos and de Vos, 2012; Wang and Kasper, 2014; Chotirmall et al., 2017; Zhu et al., 2020). This potential link is underscored by studies indicating the influence of gut health on the regulation of breathing, as well as evidence that inflammation or injury, though distal from the brain, can exert significant yet indirect impacts on respiratory control mechanisms (Wypych et al., 2019; Chunxi et al., 2020). However, it is unclear if microbiome dysbiosis is a symptom or cause of injury or disease in the nervous system leading to respiratory dysregulation (Faner et al., 2017).

Generally, two approaches are taken to study the absence and role of the microbiome in mice. First, germ-free (GF) mice are delivered via C-section into a sterile environment or embryo transfer into a GF host and, if successfully raised, are thought to be devoid of nearly all microorganisms (bacteria, viruses, and fungi). This approach is time-consuming and requires extensive infrastructure and expertise. A simpler contrasting approach utilizes broad-spectrum antibiotics to deplete the gut bacteria-only microbiome while effects on lung, urogenital, and skin biomes are not always considered. As these antibiotics are often given *ad libitum*, dosing and potentially toxic side effects are not fully considered (van Hal et al., 2013; Bayer et al., 2019). The contrast between these two approaches is fully explored in several reviews (Kennedy et al., 2018).

In the pursuit of clarity, GF mouse models—organisms born free of microorganisms without antibiotic treatment—become invaluable. These models offer a controlled window into the microbiome-brain axis, allowing us to understand the nuances of microbial contributions to adult health and disease. Further enriching this exploration, we introduce novel findings on neonate autoresuscitation—a critical reflex with implications for survival following life-threatening events, like those hypothesized to initiate Sudden Unexpected Infant Death (SUID). To understand the relationship between respiratory function and microbiome, we measured respiratory parameters at baseline and in response to three different gas challenges at two ages in GF mice and specific pathogen-free (SPF) control mice (with a normal microbiome). We hypothesized that the lack of a microbiome has minimal effect on respiratory physiology.

Using GF mouse models, whole-body barometric plethysmography in adults, and pneumotachographs in neonates, we found several differences in baseline and recovery cardiorespiratory parameters among the neonates and significant differences in body weight between neonates on postnatal days 7–8 (P7-8) and adults 6–15 weeks of age. Despite these differences, there was no significant impact on the overall survival of the neonates following repetitive anoxic gas challenges, i.e., autoresuscitation.

To further interrogate the specific contribution of the gut microbiome, we performed fecal material transplants (FMTs) in adult mice following their initial plethysmography experiment. We found no notable respiratory changes in GF *versus* SPF adult mice before or after fecal transplant across all groups. However, GF adult mice exhibited changes in oxygen consumption, indicating a potential shift in metabolic demand. These findings suggest that gut dysbiosis may not significantly influence respiratory-specific outcomes but may influence metabolic demand.

An altered microbiome could arguably be considered a disease itself. Whether gut health is a standalone disease or a symptom, our results provide an essential foundation to argue that GF mice should be strongly considered for modeling the microbiome-brain axis in health and disease. The results of this model shed new light on the relationship between microbiome and respiratory health and disease.

## 2 Materials and methods

### 2.1 Ethical approval

The Baylor College of Medicine Institutional Animal Care and Use Committee approved the animal studies under protocol AN-6171.

### 2.2 Breeding and maintenance of mice

Germ-free (GF) C57Bl/6J mice were obtained from the National Gnotobiotic Rodent Resource Center (Chapel Hill, NC) and maintained and bred in the Gnotobiotics Core animal facility at Baylor College of Medicine (Houston, TX). GF mice were maintained in positive pressure, flexible film isolators with high-efficiency particulate-filtered air and provided with irradiated food and water. GF status was assessed through mouse and isolator surface cultures following every isolator opening and monthly 16S qPCR testing of GF mouse feces. Specific pathogen-free (SPF) C57Bl/6J mice were obtained from Jackson Laboratory (Bar Harbor, ME) and bred and maintained at Baylor College of Medicine in a high-level barrier facility. SPF mice were maintained in Tecniplast individually ventilated cages until study use and had access to food and water *ad libitum*.

### 2.3 Experimental study design

#### 2.3.1 General

**FIGURE 1:**
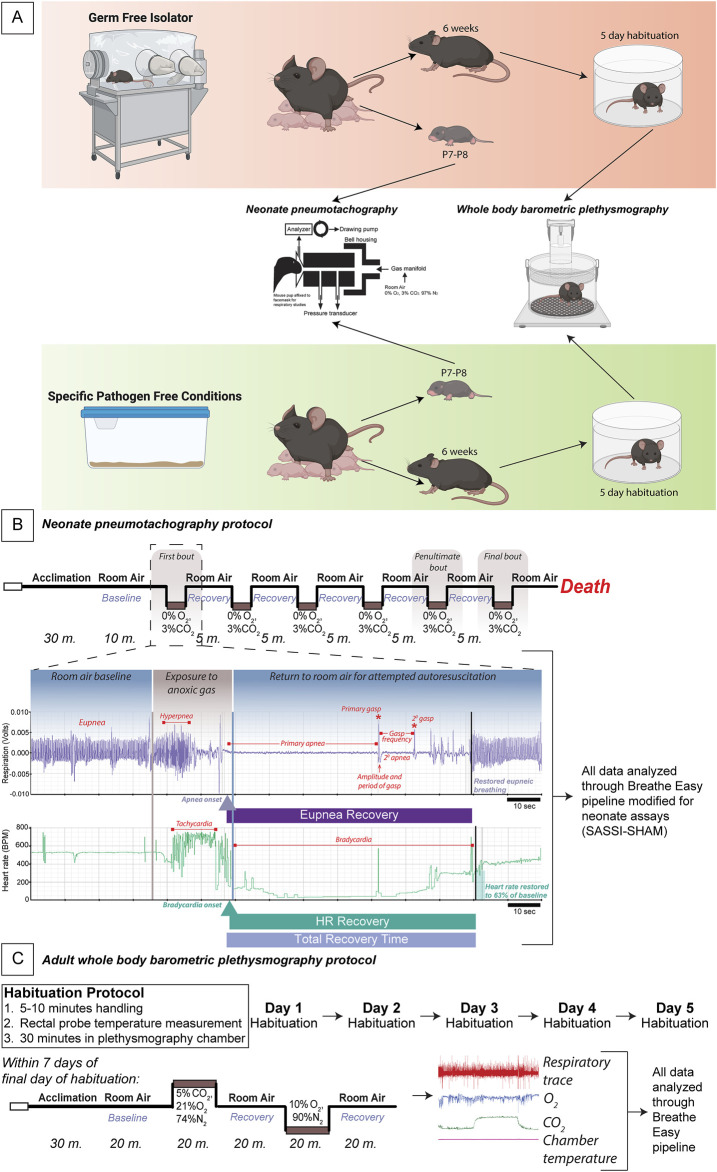
Experimental protocol for adult and neonate respiratory measurements in GF vs. SPF mice. Schema of the germ-free (GF) and specific pathogen-free (SPF) experimental protocol wherein GF and SPF pups are taken at P7-8 for pneumotachography recordings during the autoresuscitation assay and 6-7-week-old adults begin a week of habituation followed by whole-body barometric plethysmography recordings **(A)**. A schematic of the exact timeline of the neonate autoresuscitation assay **(B)** with an example trace of one single bout of autoresuscitation. All data were analyzed using a modified version of the Breathe Easy pipeline developed for neonate pneumotachography. An elaborated timeline of habituation and the hypercapnic and hypoxic challenges to assay the chemoreflex in the adult mice is shown where breathing was recorded using plethysmography **(C)**. All data were analyzed using our published waveform analysis software, Breathe Easy. Abbreviations: GF, germ-free; SPF, specific pathogen-free; P, postnatal day.

An outline of our experimental paradigms, including data analysis, is outlined in Figure 1. No animals were excluded due to pain, illness, or suffering. Randomization was not part of this study as no treatment was given, and the mice compared were lines raised in different housing conditions. However, several litters were used to complete this study to eliminate litter effects that may have existed in the mice. There was no blinding to the group as the location of the mice before the experiment indicated which group the mice belonged to. However, our automated analysis pipeline removes any bias of knowing the group, as all breath and heartbeat selections were completed using software rather than manually interrogating the files. Finally, while all animals were included in the analysis by Breathe Easy, not all parts of the recordings contained sufficient quality breathing to create outputs for each gas period. This excludes the data from those animals in the final analyses presented here. All adult mice were humanely euthanized following our Institutional Animal Care and Use Committee (IACUC) approved protocol, where each mouse was given an overdose of pharmaceutical-grade euthanasia agent containing sodium pentobarbital at a lethal dose of 390 mg/kg administered intraperitoneally. Death was the endpoint for the neonate experiments, and there are no alternative approaches for studying this outcome. Our neonate protocol has been reviewed and approved by BCM IACUC.

#### 2.3.2 Neonate face mask pneumotachography

Our neonate pneumotachography experiment is performed on postnatal day (P)7-8 mice and does not include habituation. P7-8 GF and SPF mice were taken from their home cage and immediately assessed in our neonate face mask pneumotachography assay (Figures 1A, B).

12 SPF control pups (4 females and eight males) and 9 GF pups (5 females and four males) were assessed using neonate face mask pneumotachography. Sample sizes were determined based on previous publications finding differences in survival in this assay (Chen et al., 2013; Cummings et al., 2013; Cummings, 2014).

#### 2.3.3 Adult whole-body plethysmography

Before experimental habituation, 6-7-week-old SPF mice were transferred to isolators to control for housing-related effects. 6-7-week-old GF mice were sterilely transferred to study isolators before habituation procedures. Glass plethysmography chambers were autoclaved in supply cylinders and entered into the isolator. A modified ring stand was used to hold the chamber in the appropriate orientation (Figure 1A). Mice were habituated for 5 days using our established habituation protocol (Martinez et al., 2019; Lusk et al., 2022; 2023; Chang et al., 2023) before exit and subsequent breathing measurements. Briefly, mice are handled for 5–10 min, their rectal temperature is taken, and then they are placed in the glass plethysmography chamber for 30 min daily for 5 days. Mice were then assessed using our whole-body plethysmography approach within 1 week of the final habituation day (Figure 1C).

28 SPF control mice (14 females and 14 males) and 24 GF mice (12 females and 12 males) were recorded for the initial round of adult whole-body plethysmography at 7–10 weeks of age. From these groups, 12 SPF controls (6 females and six males) and 11 GF mice (5 females and six males) received fecal material transplants (FMT) the day after their first plethysmography experiment. They were assayed a second time 3–7 weeks after the FMT at 11–15 weeks of age (Turnbaugh et al., 2009; Staley et al., 2017; Bokoliya et al., 2021). The sample size was determined using previously published work from our lab, where significant respiratory differences were found, with sample sizes from 13 to 16 per group, excluding sex (Martinez et al., 2019). However, some groups were limited due to the availability of GF mice.

### 2.4 Fecal transplant

Approximately 525 mg of fresh feces from donor mice were collected into sterile 1.7 mL snap cap tubes. 1 mL of sterile PBS was added, and tubes vortexed for 5 min to create a fecal slurry. Tubes were centrifuged at 200 *g* for 5 min. The supernatant was removed to a separate tube. 0.2 mL fresh fecal preparation was administered to mice via oral gavage using a 22-gauge gavage needle. 11 GF mice and 12 SPF mice received fecal material transplants.

### 2.5 Respiratory studies

#### 2.5.1 Neonate face mask pneumotachography

Face mask pneumotachography was carried out on P7-P8 conscious, restrained mice (Chen et al., 2013; Cummings et al., 2013). Pups were removed from their home cage and maintained on a heated blanket until they were weighed and attached to an air-tight face mask (Sun et al., 2017). Pups were then placed in a temperature-controlled (water jacketed at 36°C) chamber and given at least 10 min to acclimate in room air (21% O_2_/79% N_2_). Following acclimation, the gas was switched to an anoxic mixture (3% CO_2_/77% N_2_), which the pup was exposed to until apnea. Following the apnea, the gas was switched back to room air, and the pup was allowed to recover. Five minutes after the start of the recovery period, defined by the initial gasp following breathing and heartbeat cessation, another anoxic challenge was administered. This cycle was repeated until the pup failed to recover. Breathing and heart rate were recorded after acclimation for baseline and before and during all challenge and recovery periods. For all gas exposures, the flow rate was maintained at 12.5 mL/min.

#### 2.5.2 Whole-body plethysmography

Whole-body flow through barometric plethysmography was carried out on 7–9-week-old conscious, unrestrained mice. Within 1 week of the final habituation day, mice were removed from their home cage, weighed, and had their temperature taken rectally. They were then placed in a flow-through, temperature-controlled (water jacketed at 30°C) plethysmography chamber and given at least 20 min to acclimate in room air (21% O_2_/79% N_2_). Following acclimation, a baseline of at least 20 min was recorded in room air. Following the baseline, the chamber gas was switched to a hypercapnic mixture (5% CO_2_/21% O_2_/74% N_2_). After 20 min of hypercapnic exposure, the chamber gas was switched back to room air for another 20 min for recovery. Following the second room air exposure, the chamber gas was switched to a hypoxic mixture (10% O_2_/90% N_2_). After 20 min, the chamber gas was again switched back to room air. The final room air exposure lasted another 20 min. Immediately after the final room air exposure, mice were removed from the plethysmography chamber, their temperature was taken rectally, and they were returned to their home cage. Breathing was recorded during all three room air exposures as well as the hypercapnic and hypoxic exposures. For all gas exposures, the flow rate was 198 mL/min.

### 2.6 Data collection

#### 2.6.1 Neonate face mask pneumotachography

Pneumotach pressure changes were measured with a Validyne DP45 differential pressure transducer and CD15 carrier demodulator. The chamber temperature was continuously monitored with a ThermoWorks MicroThermo two probe. All signals were recorded in real-time with LabChartPro.

#### 2.6.2 Whole-body plethysmography

Plethysmography chamber pressure changes were measured and compared to an empty reference chamber with a Validyne DP45 differential pressure transducer and CD15 carrier demodulator. The concentration of CO_2_ in the plethysmography chamber was measured using an AEI Technology carbon dioxide sensor P-61B and AEI Technology carbon dioxide analyzer CD-3A. The concentration of O_2_ in the chamber was measured using an AEI Technology oxygen sensor N-22M and oxygen analyzer S-3A/I. The chamber temperature was continuously monitored with a ThermoWorks MicroThermo two probe. All signals were recorded in real-time with LabChartPro.

### 2.7 Data analysis and statistics

#### 2.7.1 General

Linear mixed effects models (LMEM) with a Tukey Honestly Significant Difference (HSD) post-hoc test were used for all statistical analyses. As described in our previous publication, the LMEM does not require strict adherence to assumptions (Schielzeth et al., 2020; Lusk et al., 2023). That said, we did analyze QQ and residual plots for all outcomes with three transformations of the data (raw, log_10_, and square root) to address adherence to assumptions.

#### 2.7.2 Neonate face mask pneumotachography

Data analysis was completed using an extended version of *Breathe Easy* (Lusk et al., 2023). Briefly, Signal Analysis Selection and Segmentation Integration (SASSI) interrogated each mouse’s breathing and heart rate traces and recorded all raw values extracted through the recording. Following SASSI, the extension, Selection Helper for Autoresuscitation Measurements (SHAM), calculated respiratory rate, tidal volume, ventilation, and heart rate during baseline, the number of episodes survived, induction time required for apnea, time until first gasp after apnea (gasp latency), gasp frequency between first and second gasp, and time for recovery of ventilatory frequency and heart rate to 50% and 63% of baseline for every anoxic challenge.

Following the quantification of these parameters, custom R scripts were used to perform linear mixed-effects models with a Tukey HSD *post hoc* to test for significant differences between GF and SPF pups. A *p*-value of <0.05 was used to indicate statistical significance. Individual data points on each graph mark each parameter’s average value for every pup. The mean and standard error for each pup is also included. Every mouse was included in the analysis.

#### 2.7.3 Whole-body plethysmography

Data analysis was completed using Breathe Easy, comprised of SASSI and STAGG software (Lusk et al., 2023). Briefly, SASSI interrogated the breathing trace for every mouse and identified “calm” breaths. For these breaths, SASSI determined respiratory rate (V_f_), tidal volume (V_T_), minute ventilation ( $\dot{V}$ _E_), oxygen consumption ( $V_{O_{2}}$ ), and minute ventilation normalized to oxygen consumption ( $\frac{\dot{V_{E}}}{V_{O_{2}}}$ ). SASSI also identified sighs and apneas. Sighs were identified as breaths with an amplitude at least two times as large as the average breath. Apneas were identified as instances where the interbreath interval (IBI) was at least twice as long as the average IBI.

For every parameter, STAGG used a linear mixed effects model with a Tukey Honestly Significant Difference (HSD) correction to test for significant differences between GF and SPF mice and pre- and post-fecal transplants. A *p*-value of <0.05 was used to define statistical significance, which is indicated on the graphs with an asterisk. Every graph includes individual data points for the average value of each parameter for every mouse. Error bars indicate the standard deviation of all breaths included in the analysis, and circles highlight the average of all breaths included in the analysis. However, because the data distribution includes all breaths (hundreds of thousands of data points), the error bars and averages may not always align well with the overall average points for each mouse. Every mouse was included in the analysis, but some mice were excluded from specific outcomes or challenge conditions as the quality of their recordings for that condition did not meet the criteria. As such, the final results presented here include 28 SPF control mice pre-FMT, 21 GF mice pre-FMT, 11 SPF control mice post-FMT, and 10 GF mice post-FMT.

## 3 Results

**FIGURE 2:**
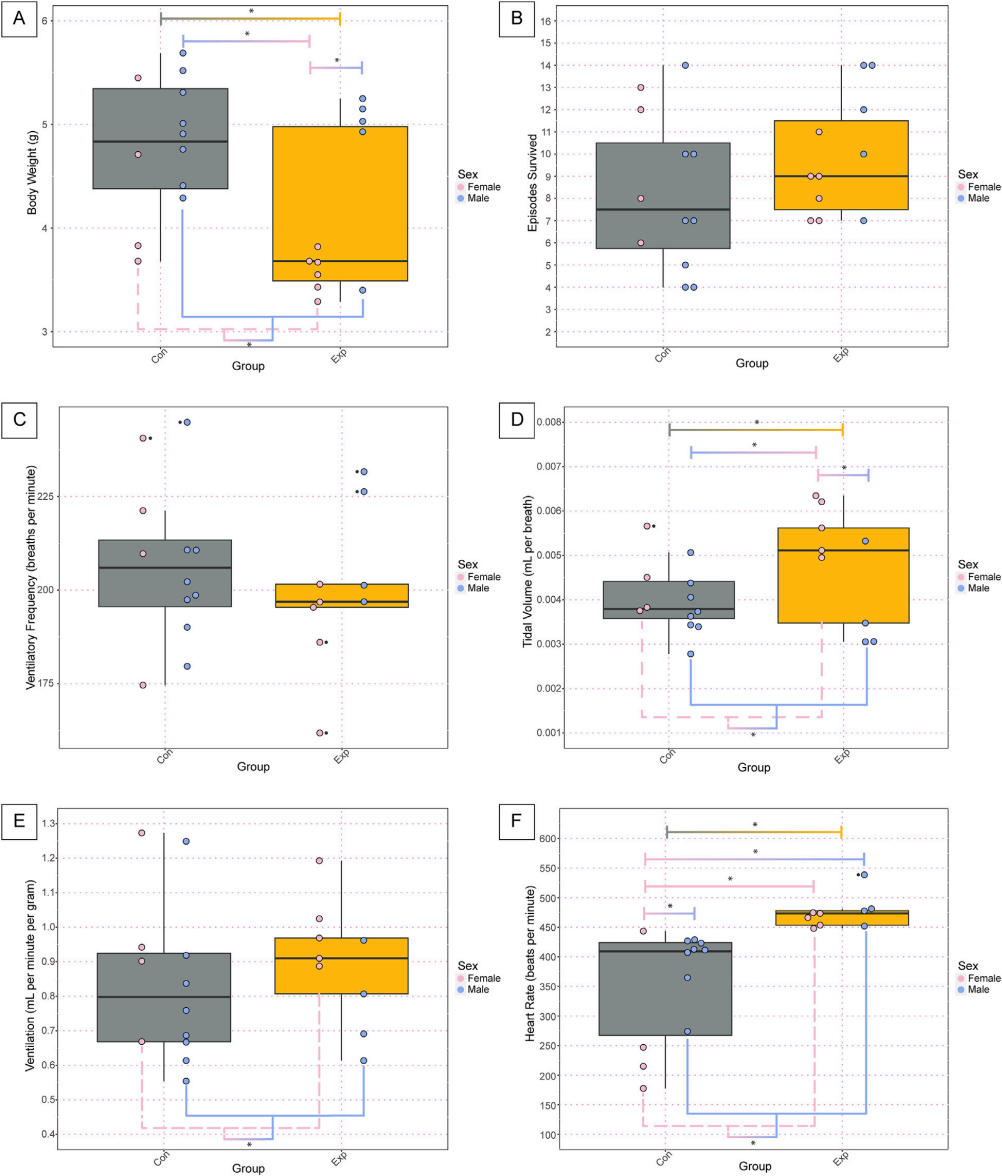
Body weight and basic cardiorespiratory parameters are altered in GF neonates at baseline. The body weight **(A)**, total number of anoxic episodes survived **(B)**, and baseline values for ventilatory frequency **(C)**, tidal volume **(D)**, ventilation **(E)**, and heart rate **(F)** for the neonate pups. Each dot represents one animal where the dot color indicates the sex (pink = female; blue = male), the boxplot fill corresponds to the group (gray = control, SPF mice; orange = experimental, GF mice), and small black dots next to data points identify outliers. Significance is defined by a *p*-value of <0.05 as determined with a linear mixed effects model and a Tukey Honest Significant Difference *post hoc* test. Exact *p*-values can be found in the text for significant differences. Comparisons are indicated with the lines connecting the two groups that were significantly different. Abbreviations: Con, control; Exp, experimental; g, grams.

We found a significant difference in body weight at P7-8 between GF (4.11g ± 0.79) and SPF control (4.80g ± 0.65) mice (*p* = 0.010, Figure 2A). This difference was primarily driven by the female GF (3.57g ± 0.19) mice, which weighed significantly less than male GF (4.75g ± 0.77, *p* = 0.015) and male SPF (4.99g ± 0.50, *p* = 0.0012) mice. Additionally, there was an overall effect of sex where female (3.91g ± 0.66) mice weighed significantly less than male (4.90g ± 0.60, *p* = 0.0027) mice. Despite the weight disparities, we found no differences in the number of bouts of successful autoresuscitation between the two groups or between sexes (Figure 2B).

In analyzing the baseline physiology of the neonates (i.e., room air breathing before the onset of the anoxic challenges), we found no significant differences in ventilatory frequency (Figure 2C). However, we found the same significant differences as with body weight for tidal volume wherein there were statistically significant differences between groups (GF: 0.0048 ± 0.0013, SPF: 0.0040 ± 0.00078, *p* = 0.04), sexes (Female: 0.005 ± 0.0009, Male: 0.0038 ± 0.0008, *p* = 0.004), SPF control males and GF females (SPF M: 0.0038 ± 0.0007, GF F: 0.0056 ± 0.0006, *p* = 0.004), and GF males and GF females (GF M: 0.0037 ± 0.001, GF F: 0.0056 ± 0.0006, *p* = 0.01) (Figure 2D). Interestingly, when considering ventilation, which is the product of ventilatory frequency and tidal volume, we only see the difference between the sexes preserved (Female: 0.97 ± 0.2, Male: 0.78 ± 0.2, *p* = 0.05) with no difference between groups or any other comparisons (Figure 2E). Additionally, we saw several significant differences in the heart rate at baseline. Specifically, we saw statistically significant differences in the heart rate between groups (GF: 473.9 ± 27, SPF: 352.7 ± 96, *p* = 0.0004), sexes (Female: 377.6 ± 125, Male: 424.9 ± 65, *p* = 0.02), female and male SPF controls (Female: 270.7 ± 119, Male: 393 ± 52, *p* = 0.02), GF males and SPF females (GF Males: 487.3 ± 37, SPF Females: 270.7 ± 119, *p* = 0.0007), and GF females and SPF females (GF Females: 463.1 ± 12, SPF Females: 270.7 ± 119, *p* = 0.001) (Figure 2F).

**FIGURE 3:**
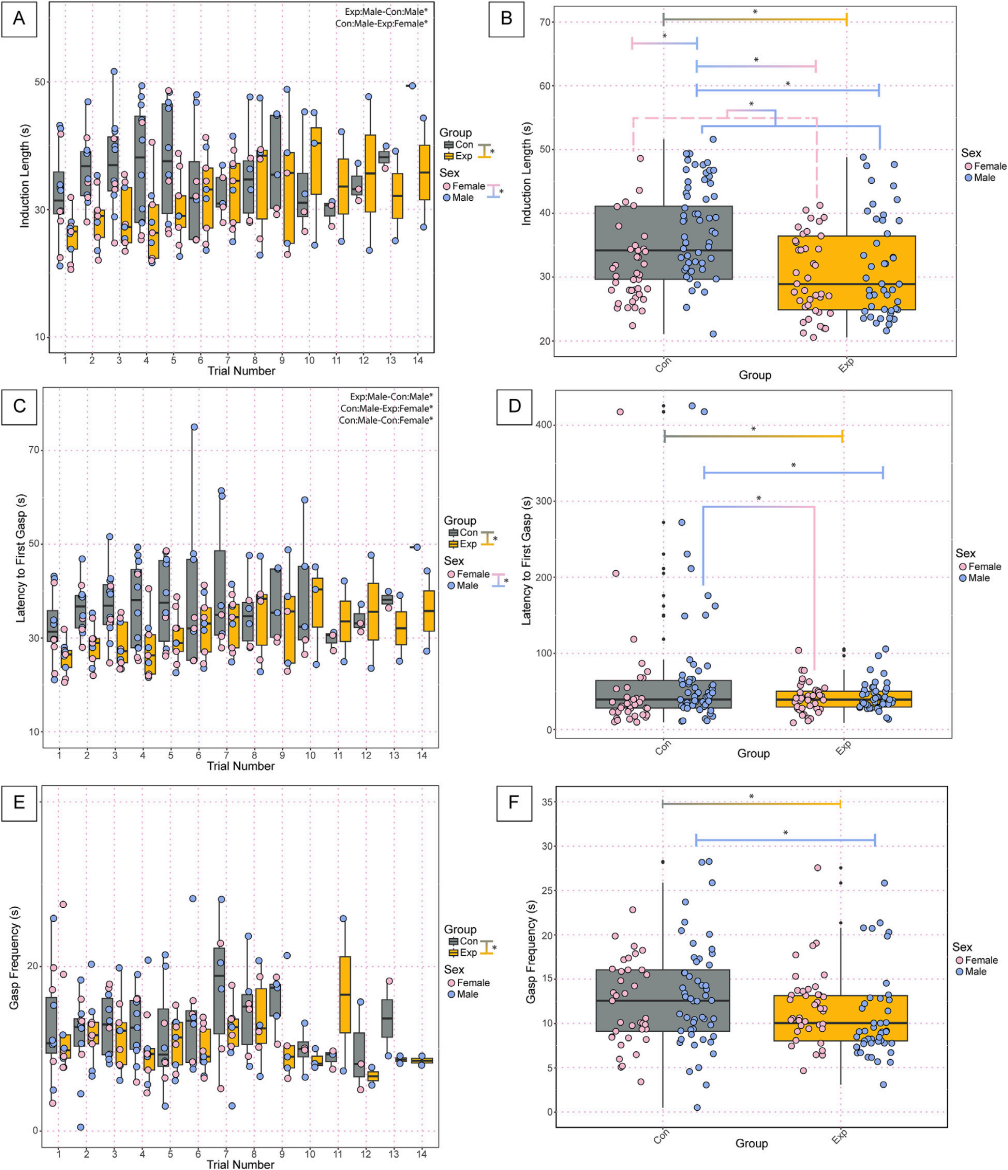
Cardiorespiratory dynamics during anoxic challenges and recovery following autoresuscitation are altered in neonatal GF *versus* control SPF mice. Induction length **(A)** is the time in seconds of exposure to an anoxic challenge required to induce apnea for each trial. Each point represents one bout of autoresuscitation in 1 mouse per trial. The average induction length among all trials is represented in **(B)**. Latency to first gasp **(C)** is the time in seconds from the onset of apnea until the first gasp, with the average latency to first gasp among challenges depicted in **(D)**. Gasp frequency **(E)** is the time in seconds between the first and second gasp and the average gasp frequency among all trials is depicted in **(F)**. In A-F, each dot represents one bout of autoresuscitation where the dot color indicates the sex (pink = female; blue = male), and the boxplot fill corresponds to the group (gray = control, SPF mice; orange = experimental, GF mice), and small black dots next to data points identify outliers. Significance indicates a *p*-value of <0.05 as determined with a linear mixed effects model and a Tukey Honest Significant Difference *post hoc* test; exact *p*-values can be found in the text for significant differences. Comparisons are indicated with the lines connecting the two groups that were significantly different. Abbreviations: Con, control; Exp, experimental; s, seconds.

In analyzing the physiology of the neonates following anoxic gas challenges, we found significant differences in various parameters. Analysis of the induction time required for apnea (termed induction length) during the anoxic challenges revealed significant differences between groups (GF: 30.9 ± 7, SPF: 36.4 ± 9, *p* = 4.02 × 10^−5^) and sexes (Female: 30.8 ± 6, Male: 36.5 ± 10, *p* = 0.000171) (Figure 3A). Assessment of average induction length among all challenges revealed significant differences between groups (GF: 30.9 ± 7, SPF: 36.4 ± 9, *p* = 1.66 × 10^−05^), sexes (Females: 30.8 ± 6, Males: 36.5 ± 10, *p* = 8.26 × 10^−05^), male and female SPF controls (Female SPF: 31.5 ± 6, Male SPF: 39.6 ± 9, *p* = 3.22 × 10^−05^), SPF males and GF females (GF Females: 30.4 ± 6, SPF Males: 39.6 ± 9, *p* = 3.63 × 10^−07^) and GF males and SPF males (GF Males: 31.5 ± 8, SPF Males: 39.6 ± 9, *p* = 1.78 × 10^−05^) (Figure 3B). Assessment of gasp latency during anoxic challenges revealed significant differences between groups (GF: 42.0 ± 19, SPF: 65.9 ± 81, *p* = 4.87 × 10^−06^), sexes (Females: 45.3 ± 52, Males: 61.5 ± 68, *p* = 4.39 × 10^−05)^, male and female SPF controls (SPF Females: 50.5 ± 71, SPF Males: 76.3 ± 87, *p* = 3.84 × 10^−06^), SPF males and GF females (GF Females: 40.9 ± 20, SPF Males: 76.3 ± 87, *p* = 1.78 × 10^−07)^, and SPF and GF males (SPF Males: 76.3 ± 87, GF Males: 43.0 ± 19, *p* = 1.86 × 10^−06^) (Figure 3C). We found a significant difference in the average gasp latency among challenges between groups (GF: 42.0 ± 19, SPF: 65.9 ± 81, *p* = 0.0085), GF females and SPF males (GF Females: 40.9 ± 20, SPF Males: 76.3 ± 87, *p* = 0.024), and GF males and SPF males (GF Males: 43.0 ± 19, SPF Males: 76.3 ± 87, *p* = 0.031) but no differences between sexes or any other comparisons (Figure 3D). Assessment of gasp frequency during anoxic challenges revealed significant differences between groups (GF: 11.2 ± 5, SPF: 13.3 ± 7, *p* = 0.047) but no differences between sexes or any other comparisons (Figure 3E). Furthermore, there was a significant difference between groups in average gasp frequency among challenges (GF: 11.2 ± 5, SPF: 13.3 ± 6, *p* = 0.040) and between GF and SPF males (GF: 10.7 ± 5, SPF: 13.7 ± 7, *p* = 0.047) but no significant differences between sexes or any other comparisons (Figure 3F).

**FIGURE 4:**
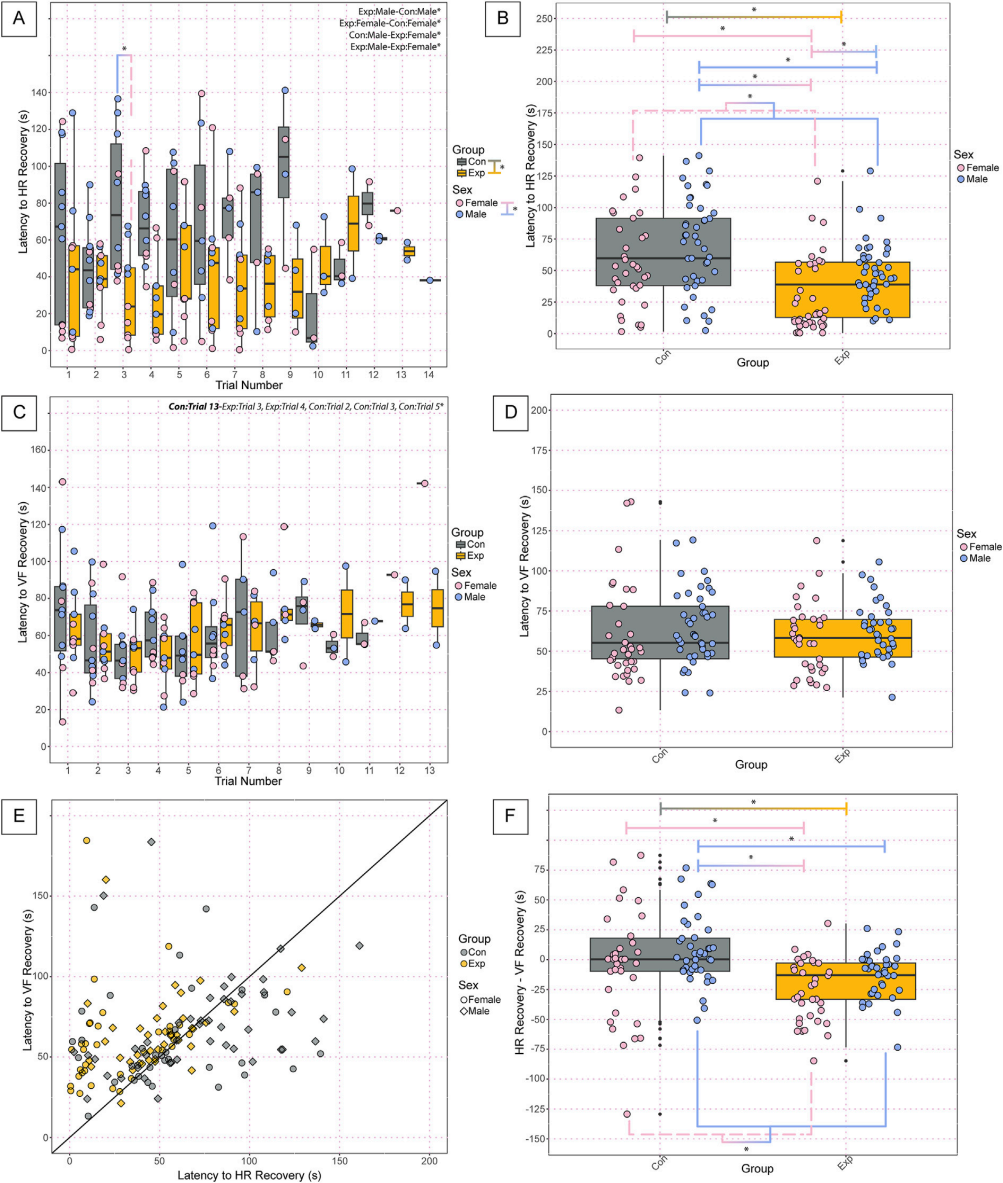
Neonatal GF mice display a shorter latency to HR recovery and decoupling in latency to HR *versus* VF recovery compared to SPF controls following anoxic challenges. Latency to heart rate recovery **(A)** by trial and the average latency to HR recovery among all challenges **(B)** indicates the time in seconds required to reach 63% of baseline values. Latency to ventilatory frequency recovery by trial **(C)** and the average latency to VF recovery among all challenges **(D)** indicate the time in seconds required to reach 50% of baseline values. The relationship between cardiovascular and ventilatory recovery is shown in **(E)**, where the time required for HR recovery is used as the x-value, and the time required for VF recovery is used as the y-value. The difference in latency to HR recovery and latency to VF recovery is depicted in **(F)** to assess decoupling effects. In **(A-D, F)**, each dot represents one bout of autoresuscitation where the dot color indicates the sex (pink = female; blue = male), the boxplot fill corresponds to the group (gray = control, SPF mice; orange = experimental, GF mice), and small black dots next to data points identify outliers. In **(E)**, each dot represents one recovery period from one animal where the dot color indicates the group (gray = control, SPF mice; orange = experimental, GF mice), and the shape of the point indicates the sex (circles = female; squares = males). Significance indicates a *p*-value of <0.05 as determined with a linear mixed effects model and a Tukey Honest Significant Difference *post hoc* test; exact *p*-values can be found in the text for significant differences. Comparisons are indicated with the lines connecting the two groups that were significantly different. Abbreviations: HR, heart rate; VF, ventilatory frequency; Con, control; Exp, experimental; s, seconds.

Assessment of cardiorespiratory recovery parameters of the neonates following anoxic gas challenges revealed several significant differences. Assessment of the latency to HR recovery during anoxic challenges revealed significant differences between groups (GF: 39.4 ± 28, SPF: 72.2 ± 65, *p* = 2.87 × 10^−06^), sexes (Females: 49.0 ± 53, Males: 60.8 ± 37, *p* = 0.000826), SPF and GF females (GF Females: 28.9 ± 30, SPF Females: 68.2 ± 64, *p* = 0.0047), SPF males and GF females (GF Females: 28.9 ± 9, SPF Males: 75.4 ± 40.2, *p* = 1.97 × 10^−07^), and SPF and GF males (GF Males: 48.8 ± 24, SPF Males: 75.4 ± 40, *p* = 0.0048) (Figure 4A). We found significant differences in the average latency to HR recovery among challenges between groups (GF: 39.4 ± 28, SPF: 72.2 ± 65, *p* = 1.11 × 10^−06^), sexes (Females: 48.0 ± 53, Male: 60.8 ± 37, *p* = 0.00054), GF females and SPF male controls (GF Females: 28.9 ± 30, SPF Males: 75.4 ± 41, *p* = 4.50 × 10^−08^), GF females and SPF females (GF Females: 28.9 ± 30, SPF Females: 68.2 ± 64, *p* = 0.00178), GF males and females (GF Females: 28.9 ± 30, GF Males: 48.8 ± 24, *p* = 0.0256), and GF and SPF males (GF Males: 48.8 ± 24, SPF Males: 75.4 ± 41, *p* = 0.00524) (Figure 4B). Assessment of the latency to VF Recovery during anoxic challenges revealed significant differences between SPF trial 13 and trial 2 (SPF trial 13: 475.9, SPF trial 2: 56.54 ± 25, *p* = 0.049), SPF trial 13 and trial 3 (SPF trial 13: 475.9, SPF trial 3: 50.6 ± 18, *p* = 0.0272), SPF trial 13 and GF trial 3 (SPF trial 13: 475.9, GF trial 3: 48.8 ± 14, *p* = 0.0217), SPF trial 13 and GF trial 4 (SPF trial 13: 475.9, GF trail 4: 48.4 ± 14, *p* = 0.0204), SPF trial 13 and 5 (SPF trial 13: 475.9, SPF trial 5: 50.7 ± 24, *p* = 0.0323) but no significant differences between groups, sexes, nor any other comparisons (Figure 4C). There were no significant differences in the average latency to VF recovery among challenges between groups, sexes, nor any other comparisons (Figure 4D). When plotting the latency to HR recovery *versus* the latency to VF recovery as an XY plot with a line of identity to assess any decoupling effects following challenges, appreciable differences are visible in the cluster patterns between control and experimental mice. Overall, experimental GF mice have a shorter latency to HR recovery following challenges compared to SPF control mice, implicating a decoupled cardiovascular and respiratory recovery (Figure 4E). Furthermore, assessment of the average difference between latency to HR recovery and VF recovery among challenges revealed significant differences between groups (GF: −30.6 ± 61, SPF: 5.8 ± 79, *p* = 3.65 × 10^−05^), sexes (Females: −17.0 ± 49, Males: −7.1 ± 87, *p* = 0.00744), GF and SPF females (GF Females: −30.6 ± 35, SPF Females: −3.5 ± 57, *p* = 0.0206), SPF males and GF females (GF Females: −30.6 ± 35, SPF Males: 12.3 ± 91, *p* = 1.26 × 10^−05^), and SPF and GF males (GF Males: −30.6 ± 76, SPF Males: 12.3 ± 91, *p* = 0.0155) (Figure 4F). Decoupling of HR and VF recovery following autoresuscitation has been reported before and is implicated in decreased survival (Dosumu-Johnson et al., 2018). Interestingly, however, there is no difference in survival in these data. Further work is warranted to understand what role, if any, cardiorespiratory decoupling plays in autoresuscitation outcomes.

**FIGURE 5:**
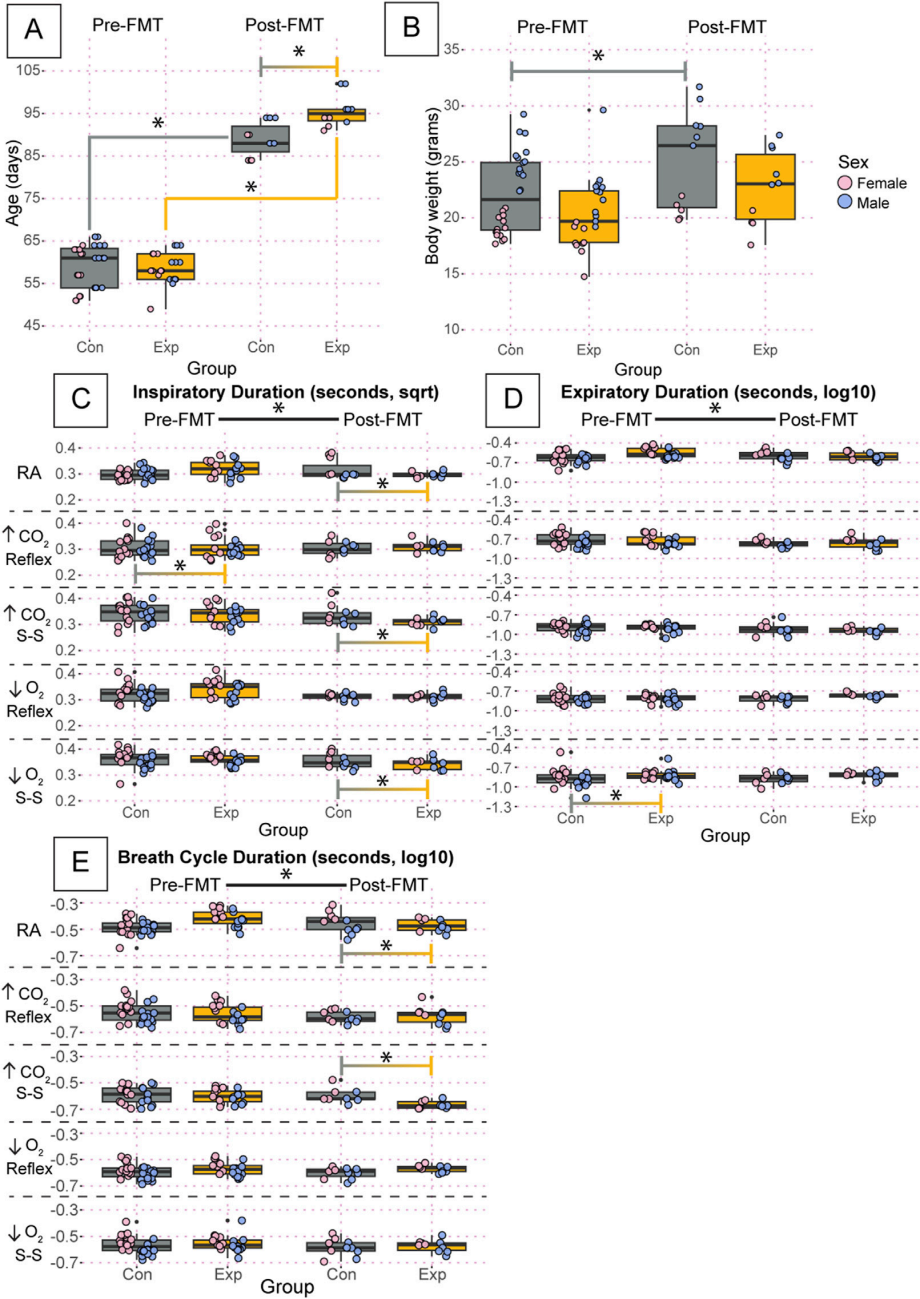
Despite small differences in age and body weight, there are no group-driven differences in respiratory outcomes between GF and control mice. Age **(A)** and body weight **(B)** of the adult mice studied using whole-body barometric plethysmography where controls are specific pathogen-free and experimental are germ-free. Inspiratory duration **(C)**, expiratory duration **(D)**, and breath cycle duration **(E)** for each animal during room air, the initial hypercapnic reflex, steady-state hypercapnia, initial hypoxic reflex, and steady-state hypoxia are shown. Each dot is the average value for every quality breath in each gas condition as determined by Breathe Easy. Error bars show the standard deviation of all breaths. Data for age and weight are raw values, whereas inspiratory duration is square root transformed, and expiratory duration and breath cycle duration are log10 transformed. Asterisks indicate significance with a *p*-value <0.05 as determined by a linear mixed effects model with a Tukey Honest Significant Difference *post hoc* test. Exact *p*-values can be found in the text for significant differences. The control group is indicated by gray boxes, and the experimental group is indicated by orange boxes. Pink dots indicate females and blue dots indicate males. Pre-FMT indicates values before the fecal transplant and Post-FMT after. Abbreviations: ↑CO_2_, 5% carbon dioxide (hypercapnia); ↓O_2_, 10% oxygen (hypoxia); Con, control; Exp, experimental; sqrt, square root; Pre-FMT, pre-fecal material transplant; Post-FMT, post-fecal material transplant; S-S, steady-state.

There was a significant difference in the age of the adult mice post-fecal material transplant (FMT) (GF: 95.6 ± 3.8, SPF: 88.9 ± 3.9, *p* = 2.3 × 10^−5^), but not pre-FMT (Figure 5A). We also found a significant increase in body weight in control SPF mice before and after FMT (Pre: 22.1 ± 3.4, Post: 25.1 ± 4.5, *p* = 0.0006) but not in GF mice (Figure 5B).

We found significant changes in inspiratory, expiratory, and breath cycle duration when comparing values before and after FMT (Figures 5C–E). Values for inspiratory duration are reported as square root transformed, expiratory duration as log10 transformed, and breath cycle duration as log10 transformed. Particularly for inspiratory duration, both GF and SPF control mice showed a significant change following FMT for every condition (Figure 5C). Interestingly, expiratory duration only showed changes in both groups in room air, hypercapnia steady state, and hypoxia steady state. During the initial hypercapnic reflex and hypoxic reflex, only the controls and GF mice showed significant changes following FMT, respectively. This trend carried forward into the breath cycle duration, where both groups showed significant changes following FMT in room air, hypercapnic steady state, and hypoxic steady state. Still, only the control group showed an effect of FMT in the initial hypercapnic response, and neither group showed an effect in the initial hypoxic response (Figure 5E). There was a significant difference in pre-FMT inspiratory duration during the initial hypercapnic response (GF: 0.324 ± 0.06, SPF: 0.340 ± 0.06, *p* = 0.0074) that was ameliorated following FMT (Figure 5C). Additionally, there was a significant difference in expiratory duration pre-FMT in GF mice compared to SPF during the hypoxic steady state (GF: −0.854 ± 0.2, SPF: −0.908 ± 0.2, *p* = 0.00012) that was also ameliorated following FMT (Figure 5D). We also found significant differences only post-FMT in inspiratory duration during room air (GF: 0.337 ± 0.06, SPF: 0.381 ± 0.08, *p* = 1.08 × 10^−10^), the hypercapnic steady state (GF: 0.316 ± 0.04, SPF: 0.340 ± 0.05, *p* = 3.1 × 10^−8^), and hypoxic steady state (GF: 0.350 ± 0.04, SPF: 0.367 ± 0.05, *p* = 5.8 × 10^−7^) (Figure 5C); and breath cycle duration during the hypercapnic steady state (GF: −0.674 ± 0.09, SPF: −0.639 ± 0.1, *p* = 1.6 × 10^−5^) (Figure 5E). Despite the changes in inspiratory and expiratory duration, only the post-FMT decrease in inspiratory duration during room air and the hypercapnic steady-state resulted in an overall change in breath cycle duration.

**FIGURE 6:**
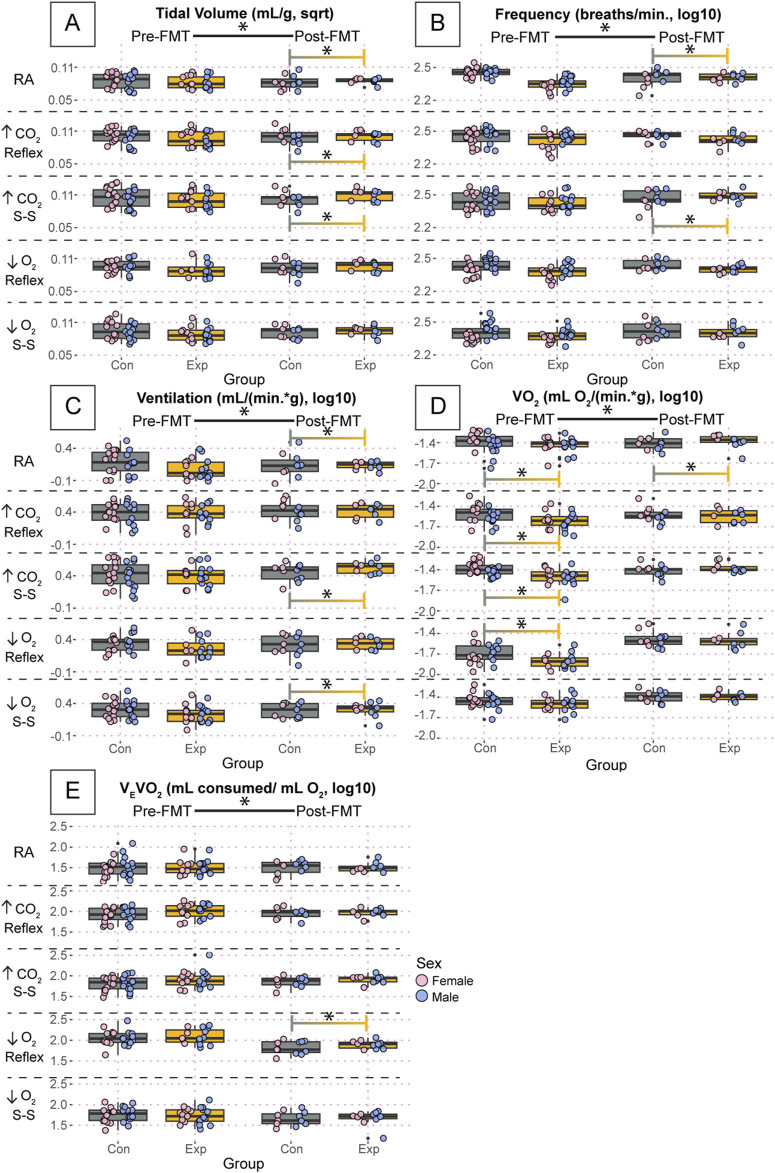
Oxygen consumption was significantly affected in GF mice, but FMT induced changes in purely respiratory outcomes. Tidal volume **(A)**, ventilatory frequency **(B)**, ventilation **(C)**, oxygen consumption **(D)**, and ventilatory equivalents of oxygen **(E)** for adult germ-free (Exp) and specific pathogen-free (Con) mice during room air, the initial hypercapnic reflex, steady-state hypercapnia, the initial hypoxic reflex, and steady-state hypoxia. Each dot is the average value for every quality breath in each gas condition as determined by Breathe Easy. Error bars show the standard deviation of all breaths. Tidal volume data are square root transformed; all other data are log10 transformed. Asterisks indicate significance with a *p*-value <0.05 as determined by a linear mixed effects model with a Tukey Honest Significant Difference *post hoc* test. Exact *p*-values can be found in the text for significant differences. Gray boxes indicate the control group and orange boxes indicate the experimental group. Pink dots indicate females and blue dots indicate males. Pre-FMT indicates values before the fecal transplant and Post-FMT after. Abbreviations: ↑CO_2_, 5% carbon dioxide (hypercapnia); ↓O_2_, 10% oxygen (hypoxia); Con, control; Exp, experimental; sqrt, square root; Pre-FMT, pre-fecal material transplant; Post-FMT, post-fecal material transplant; S-S, steady-state.

In a more refined analysis of the respiratory characteristics and metabolic measurements, we found that most variables followed the same trends as the basic breath variables (inspiratory, expiratory, and breath cycle durations) (Figure 6). For example, there were differences in pre- and post-FMT during every air condition in at least one group for tidal volume, ventilation, oxygen consumption, and ventilatory equivalents of oxygen (Figures 6A, C–E). Values for tidal volume are reported as square root transformed, and all other variables in Figure 6 are reported as log10 transformed. Most differences between groups were only present post-FMT including tidal volume during room air (GF: 0.084 ± 0.01, SPF: 0.075 ± 0.02, *p* = 0.006), the hypercapnic reflex (GF: 0.10 ± 0.01, SPF: 0.096 ± 0.02, *p* = 0.04), and the hypercapnic steady-state (GF: 0.11 ± 0.01, SPF: 0.10 ± 0.01, *p* = 0.008) (Figure 6A); ventilatory frequency during room air Post (GF: 2.18 ± 0.2, SPF: 2.22 ± 0.2, *p* = 0.009), and the hypercapnic steady-state (GF: 2.45 ± 0.09, SPF: 2.38 ± 0.1, *p* = 2.0 × 10^−5^) (Figure 6B); ventilation during room air (GF: 0.061 ± 0.2, SPF: −0.087 ± 0.3, *p* = 1.1 × 10^−5^), the hypercapnic steady-state (GF: 0.51 ± 0.2, SPF: 0.43 ± 0.2, *p* = 0.0001), and the hypoxic steady-state (GF: 0.28 ± 0.2, SPF: 0.26 ± 0.2, *p* = 0.01) (Figure 6C); and ventilatory equivalents of oxygen during the initial hypoxic reflex (GF: 1.84 ± 0.2, SPF: 1.75 ± 0.2, *p* = 0.02) (Figure 6E). Importantly, however, there were significant differences in oxygen consumption before FMT that seemed to be ameliorated post-FMT. Specifically, oxygen consumption during the initial hypercapnic reflex [Pre(GF: −1.62 ± 0.1, SPF: −1.54 ± 0.2, *p* = 0.004), Post (GF: −1.55 ± 0.1, SPF: −1.56 ± 0.1 *p* = 1)], the hypercapnic steady-state [Pre(GF: −1.49 ± 0.1, SPF: −1.41 ± 0.1, *p* = 0.006), Post (GF: −1.38 ± 0.1, SPF: −1.42 ± 0.1, *p* = 0.2)], and the initial hypoxic reflex [Pre(GF: −1.78 ± 0.1, SPF: −1.65 ± 0.2, *p* = 2.7 × 10^−10^), Post (GF: −1.53 ± 0.2, SPF: −1.48 ± 0.2, *p* = 0.98)] all showed significant differences at baseline, but not post-FMT (Figure 6D). Interestingly, the baseline difference in oxygen consumption during room air was still significantly different post-FMT [Pre(GF: −1.50 ± 0.2, SPF: −1.43 ± 0.2, *p* = 0.03), Post (GF: −1.39 ± 0.1, SPF: −1.46 ± 0.2, *p* = 0.0001)].

**FIGURE 7:**
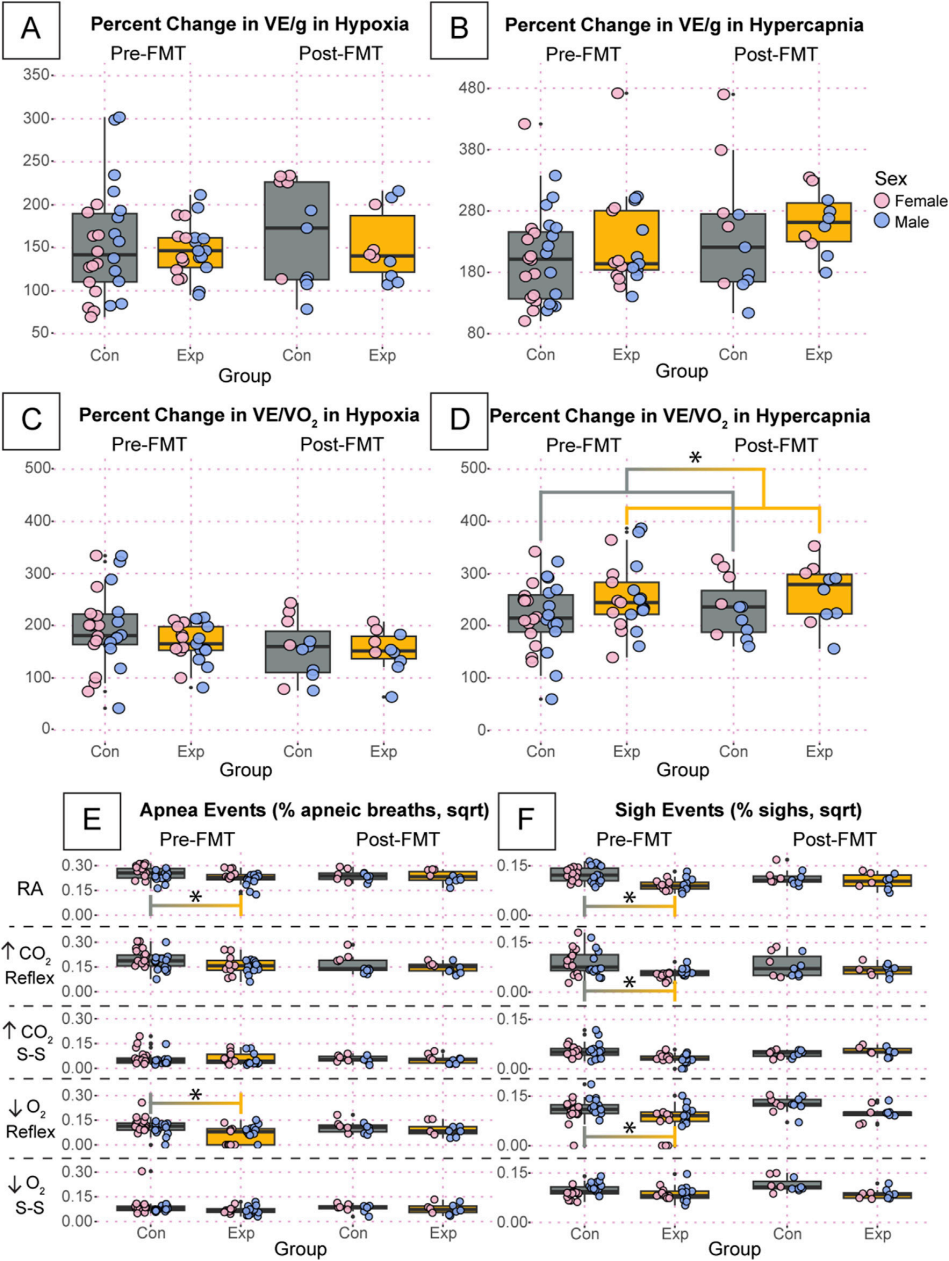
The percentage change of key respiratory variables are relatively unchanged and percent of apnea and sigh events are significantly affected in GF mice compared to SPF controls. Percent change if weight normalized ventilation during hypoxia **(A)** and hypercapnia **(B)** are shown. We also present the percent change in ventilatory equivalents of oxygen during hypoxia **(C)** and hypercapnia **(D)**. Apnea **(E)** and sigh **(F)** events per total breaths for adult germ-free (Exp) and specific pathogen-free (Con) mice during room air, the initial hypercapnic reflex, steady-state hypercapnia, the initial hypoxic reflex, and steady-state hypoxia. All apnea and sigh data are square root transformed. Asterisks indicate significance with a *p*-value <0.05 as determined by a linear mixed effects model with a Tukey Honest Significant Difference *post hoc* test. Exact *p*-values can be found in the text for significant differences. Gray boxes indicate the control group, and orange boxes indicate the experimental group. Pink dots indicate females and blue dots indicate males. Pre-FMT indicates values before the fecal transplant and Post-FMT after. Abbreviations: ↑CO_2_, 5% carbon dioxide (hypercapnia); ↓O_2_, 10% oxygen (hypoxia); Con, control; Exp, experimental; sqrt, square root; Pre-FMT, pre-fecal material transplant; Post-FMT, post-fecal material transplant; S-S, steady-state.

To further investigate potential differences in breathing that may be masked by variation in room air, we calculated the percent change in our primary respiratory variables during both hypoxia and hypercapnia where the steady-state was used as the gas exposure value, as we saw the most differences during these periods. We found no differences in weight normalized ventilation in hypoxia or hypercapnia (Figures 7A, B). There was a significant increase in the percent change of ventilatory frequency pre-FMT in the GF mice in both hypoxia (*p* = 0.002) and hypercapnia (*p* = 0.001, Supplementary Figure 1A, B). It appears that there was a non-significant reduction in the percent change of tidal volume during hypoxia (Supplementary Figure 1C), but this was not present in hypercapnia (Supplementary Figure 1D). Therefore, the change in VF must not have been large enough to cause a significant change in ventilation. We also found no difference in ventilatory equivalents of oxygen during hypoxia (Figure 7C); however, we did find a significant difference between experimental and control groups overall during hypercapnia (*p* = 0.03, Figure 7D). Because we saw no differences in oxygen consumption (Supplementary Figure 1E, F), this difference is likely driven by the change in VF.

Finally, we found significant differences in the apnea and sigh rates between GF and SPF mice during several exposures (Figures 7E, F). All values for apnea and sigh rates are square root transformed. At the pre-FMT timepoint, GF mice showed a significantly reduced apnea rate compared to SPF controls during room air (GF: 2.83 ± 0.5, SPF: 3.43 ± 0.4, *p* = 0.03) and the initial hypoxic reflex (GF: 1.03 ± 0.7, SPF: 1.72 ± 0.7, *p* = 0.003) (Figure 7E). This difference was ameliorated post-FMT. We also showed a significantly reduced sigh rate pre-FMT in GF mice compared to SPF controls during room air (GF: 1.16 ± 0.2, SPF: 1.68 ± 0.3, *p* = 0.0005), the initial hypercapnic response (GF: 0.853 ± 0.2, SPF: 1.29 ± 0.6, *p* = 0.01), and the initial hypoxic response (GF: 1.21 ± 0.6, SPF: 1.68 ± 0.3, *p* = 0.003) (Figure 7F). This difference was also ameliorated post-FMT.

## 4 Discussion

Few studies have interrogated the effects of gut dysbiosis on the control of breathing. Even fewer studies have assessed neonatal and adult mice to understand how changes in the gut microbiome may alter autonomic responses during a critical developmental period and into adulthood. Data presented here provide crucial insights into the microbiome’s role in respiratory control and provide a baseline for future studies on how gut and other microbiome dysbioses affect cardiorespiratory function and responses throughout the lifespan. Our study brings a different perspective on how an absent microbiota might interact with respiratory systems. Previous work largely focuses on antibiotic-induced microbiome depletion (O’Connor et al., 2019). While it is accepted that chronic administration of broad-spectrum antibiotics results in significant depletion of the gut bacterial microbiome while leaving viral and fungal populations largely intact (Desbonnet et al., 2015a; Hoban et al., 2016), many adverse side effects are often not fully considered, as this approach is rapid and inexpensive compared to germ-free and gnotobiotic models. For example, chronic antibiotic treatment for microbial depletion has been shown to have neurotoxic effects, result in unreliable transplantation, and promote fungal overgrowth when administered for more than 7 days (Lundberg et al., 2016; Kennedy et al., 2018; Tirelle et al., 2020). In addition to these caveats with antibiotic-treated mice, germ-free mice are seen as better models for microbial transplantation (Lundberg et al., 2016). For these reasons, we chose to use the GF mouse model to understand the role of the microbiome in breathing.

Our study revealed significant physiological differences between germ-free (GF) and specific pathogen-free (SPF) control mice, with notable effects of sex, which has been reported before in normobiotic (Yang et al., 2014; Casimiro et al., 2021) and GF mice at P23 (Clarke et al., 2013). By postnatal day 7–8 (P7-8), GF mice, particularly females, weighed significantly less than their SPF counterparts. Interestingly, by 7–10 weeks of age, the difference in weight was no longer significant. As the adults aged, only the SPF mice gained significantly more weight. Despite the GF adults being significantly older post-FMT than SPF adults, the GF adult mice post-FMT still had a lower average body weight than the SPF controls. Indeed, if age was considered a covariate in the model, then body weight in GF was significantly lower than SPF post-FMT (*p* = 0.0002). This suggests that although the body weights normalize to an extent, the SPF mice still have an advantage in gaining weight. These results are congruent with previously published work demonstrating that GF mice show muscular atrophy from decreased protein production in genes responsible for the assembly and function of neuromuscular junctions (Lahiri et al., 2019). It may also be possible that microbiota could affect satiety, thus leading to differing weights via drive to eat rather than metabolic demand. Further investigation will be necessary to illuminate these pathways.

While ages at the time of the post-FMT experiment were significantly different, body weight was considered a better proxy for developmental age, and there was no significant difference between the two groups regarding adult body weight. Additionally, we do not believe that a difference of 10 days in adult mice would make a biologically meaningful difference in the data collected. However, it would be prudent to understand at which time points the two lines match various physiological outcomes to better understand the underlying pathologies that may be driving the differences we can measure.

Neonatal GF mice displayed a higher baseline tidal volume than SPF control mice, with a female-specific increase in VT compared to SPF female controls and male GF experimental mice. Our GF neonate studies are the first of their kind; thus, we can only compare results with adult studies, where our results are in contrast with previous studies in GF mouse models that report a lower ventilatory frequency in P23 GF mice compared to controls (Barfod et al., 2023) and in studies utilizing antibiotic-depleted microbiome male-only rat models that report no differences in adult baseline respiratory measures (O’Connor et al., 2019). Although not statistically significant, the neonatal GF mice in our study showed a non-significant decrease in ventilatory frequency compared to SPF control mice. However, this may be driven by group-independent, female-specific tidal volume changes with consequential ventilation differences. Furthermore, when looking at percent change from room air, our data suggest GF mice have a greater ventilatory frequency during both hypoxic and hypercapnic challenges. The reported decrease in ventilatory frequency but no difference in other respiratory measures in prior GF studies was attributed to modulation in lung microbiome populations. Specific transcriptional changes observed at P10 were predicted to result in downstream lung morphological changes and surfactant production at P23 (Barfod et al., 2023). However, plethysmography was not completed at P10 to determine any respiratory changes, nor was lung morphological analysis or transcriptomics performed at P23 to confirm that changes at P10 may carry forward throughout development to underlie the decreased respiratory rate at P23. Differences in baseline respiratory parameters in our study may be due to female-specific decreases in body weight, as increased weight is associated with lower tidal volume and increased respiratory frequency with concomitant reductions in respiratory compliance and airway resistance (Polotsky et al., 2011; Littleton, 2020). This may also underlie the increased precent changes in ventilatory frequency we observed following gas challenges. There were also significant differences in plethysmography approaches, including habituation that could drive interacting differences across affective state, biome status, and breathing (Neufeld et al., 2011; Clarke et al., 2013; Crumeyrolle-Arias et al., 2014; Martinez et al., 2019).

Additionally, there were various differences in the baseline heart rate of neonatal GF and SPF mice; however, these observed baseline heart rate differences may be driven by differences in body weight, as noted for baseline neonatal respiratory measures. Specifically, all statistically significant differences in neonatal baseline heart rate involved the SPF control female mice where within that group, there were three females with notably lower heart rates than the fourth (3 F: 213.2 ± 35, 1 F: 443.4), which likely also perpetuated the large standard deviation in this group (SD = 119). Importantly, the three females with a lower heart rate also had lower body weights than the fourth (3 F: 4.07 ± 0.6, 1 F: 5.45). Our data suggest this relationship between body weight and baseline heart rate could be causative, wherein lower body weight produces a lower heart rate (Lopez et al., 2017; Mahgerefteh et al., 2021). Although the mechanism is unclear and not tested in these studies, we hypothesize it could be related to an immature peripheral cardiovascular system or central cardiovascular circuitry.

Though our studies utilized adult GF mice with some fundamental differences in how the respiratory assays were carried out, i.e., habituation and concurrent metabolic studies, and different measurement techniques (i.e., restrained, head out, unhabituated plethysmography), our results align well with prior studies that interrogated antibiotic depleted mice or GF mice. One notable difference is the lack of a ventilatory frequency and ventilation difference between GF and SPF mice during the hypercapnic response pre-FMT, which has been reported previously (O’Connor et al., 2019). We attribute this to differences in protocols and their utilization of an antibiotic-treated male-only rat model compared to our germ-free mouse model using both sexes. Notably, the antibiotic-treated model is expected to most effectively eliminate the gut microbiome, specifically, although some evidence in humans suggests there can be effects in other organs (Jo et al., 2021). Conversely, our model lacks microorganisms throughout the body. This is an essential difference as well when comparing the two findings. We found that all differences in baseline breathing parameters in room air were also present post-FMT.

Most significant differences between the GF and SPF groups were found after FMT. Additionally, FMT affected inspiratory duration more drastically than expiratory duration. Furthermore, the FMT process resulted in significant changes before and after the procedure in almost every condition measured, whereas differences between groups were much less frequent. Adverse effects such as esophageal trauma, peripheral nerve trauma, stress, and regurgitation followed by aspiration are not uncommon following the orogastric gavage technique and could all affect respiratory outcomes (Damsch et al., 2011; Arantes-Rodrigues et al., 2012). It is possible that mechanical disruption of neural diaphragm projections during the FMT gavage could have damaged the more active inspiratory phase of breathing while leaving the more passive expiratory phase relatively unchanged. We hypothesize that many post-FMT findings may be related to unintentional damage to the peripheral respiratory circuitry rather than true changes inflicted by an alteration in the gut-microbiome-brain axis. Alternatively, gavage-FMT may drive microbiome dysbiosis or alterations in SPF mice and establish a mal-adapted microbiome in GF mice. Future work is needed to analyze the nasal and esophageal anatomy post-FMT to determine possible contributions of the fecal material transplant, aspiration, or esophageal trauma and consider alternative inoculation approaches.

Of note, however, were the findings regarding oxygen consumption. Oxygen consumption, a proxy for metabolic rate, significantly differed between GF and SPF mice pre-FMT in room air, the initial and steady-state hypercapnic reflex, and the initial hypoxic reflex. Before FMT, GF mice showed a significantly lower metabolism than SPF mice. We hypothesize that the mouse’s overall metabolic demand is lower because there are no microbes to consume energy. Indeed, the absence of microflora results in a 20%–30% reduction in metabolic rate but shows little influence on functional respiration or oxidative phosphorylation of mitochondria (Sewell et al., 1975; Hoces et al., 2022). This difference was ameliorated post-FMT for the hypercapnic and hypoxic responses, indicating the gut microbiome’s role in regulating metabolism. Further work is warranted to more directly measure metabolism in these models, including fecal and urine production inside metabolic chambers.

In response to anoxic gas challenges, neonatal GF experimental mice displayed a decreased induction time, gasp latency, and gasp frequency and had a faster HR recovery but no change in latency to VF recovery compared to SPF control mice. There were also several sex-specific modulations in cardiorespiratory recovery parameters following anoxic gas challenges observed in our study. Regardless of experimental treatment, males had a longer induction length than females. Furthermore, GF males had a decreased gasp latency, gasp frequency, and latency to HR Recovery compared to SPF control males. GF females also displayed a decreased latency to HR recovery following anoxic gas challenges compared to SPF control females, but no differences in other parameters. Interestingly, in the adults, both apneas and sighs occurred less frequently in the GF mice than in SPF mice, a phenotype mitigated by fecal transfer. This may suggest that the lack of a microbiome increases the stability or decreases the adaptability of breathing.

Modulations in dopaminergic, serotonergic, and noradrenergic metabolites in the hippocampus, amygdala, prefrontal cortex, and brainstem of males have been reported in both GF mouse models and antibiotic-depleted microbiome rodent models (Clarke et al., 2013; Desbonnet et al., 2015b; Hoban et al., 2016; O’Connor et al., 2019). Perturbations to these monoamine systems are associated with alterations in cognition, anxiety, and autonomic function, such as cardiorespiratory control (Clarke et al., 2013; Desbonnet et al., 2015b; Hoban et al., 2016; O’Connor et al., 2019). Compared to females, male GF mice have greater perturbations to the serotonergic system both peripherally and centrally and mitigated BDNF expression, which was accompanied by alterations in anxiety-like behavior (Clarke et al., 2013). GF mice in our study may have dysregulated dopaminergic, serotonergic, or noradrenergic neurotransmission in key brainstem regions implicated in cardiorespiratory regulation in response to changes in O_2_ and CO_2_, which is more prominent in males, that underlie the observed differences in cardiorespiratory function following anoxic gas challenges and the difference in apnea and sigh rates in hypoxic and hypercapnic conditions. Future studies investigating metabolite and gene expression of these key systems in brainstem regions implicated in cardiovascular and autonomic control in GF *versus* SPF control mice will shed light on this hypothesis.

Overall, it is plausible that dysbiosis can affect respiratory outcomes. However, it is more likely that this connection is secondary or tertiary, involving altered body composition, metabolic needs, and neurochemical abnormalities. Given the non-significant changes in ventilation in the neonate groups and the difference in heart rate, it may also be possible that autonomic control of respiration and the cardiovascular system is disrupted, which could be done through the neurochemical pathways described above. This work sheds light on the possible effects of chronic antibiotic treatment on respiratory outcomes, which will be important to understand for infectious disease treatment, particularly infections that affect the respiratory tract or respiratory function. While it is clear that the lack of a microbiome changed several physiological parameters, particularly in neonates, there is not a significant effect on survival or long-term patency of the respiratory system. Further work will be necessary to understand the minutia of microorganism contributions to respiratory control and the involvement of metabolic demand in previously reported changes.

## Data Availability

The data presented in the study are deposited in the Zenodo repository, DOI: 10.5281/zenodo.12103038 (adult data) and DOI: 10.5281/zenodo.12104021 (neonate data).

## Supplementary material

SUPPLEMENTARY FIGURE S1 Percent change in respiratory variables from room air show significant changes in ventilatory frequency and weight normalized tidal volume. Percent change in ventilatory frequency **(A, B)**, weight normalized tidal volume **(C, D)**, and oxygen consumption **(E, F)** are shown during hypoxia **(A, C, E)** and hypercapnia **(B, D, F)**. Asterisks indicate significance with a *p*-value <0.05 as determined by a linear mixed effects model with a Tukey Honest Significant Difference *post hoc* test. Exact p-values can be found in the text for significant differences. Gray boxes indicate the control group, and orange boxes indicate the experimental group. Pink dots indicate females and blue dots indicate males. Pre-FMT indicates values before the fecal transplant and Post-FMT after. Abbreviations: VF, ventilatory frequency; VT/g, weight normalized tidal volume; VO_2_/g, weight normalized oxygen consumption; Con, control; Exp, experimental; Pre-FMT, pre-fecal material transplant; Post-FMT, post-fecal material transplant.
