# Supplementary figures and images for: The microbiome is dispensable for normal respiratory function and chemoreflexes in mice
Source: Front Physiol. 2024 Dec 6;15:1481394. doi: 10.3389/fphys.2024.1481394 (PMC11659286; doi:10.3389/fphys.2024.1481394)

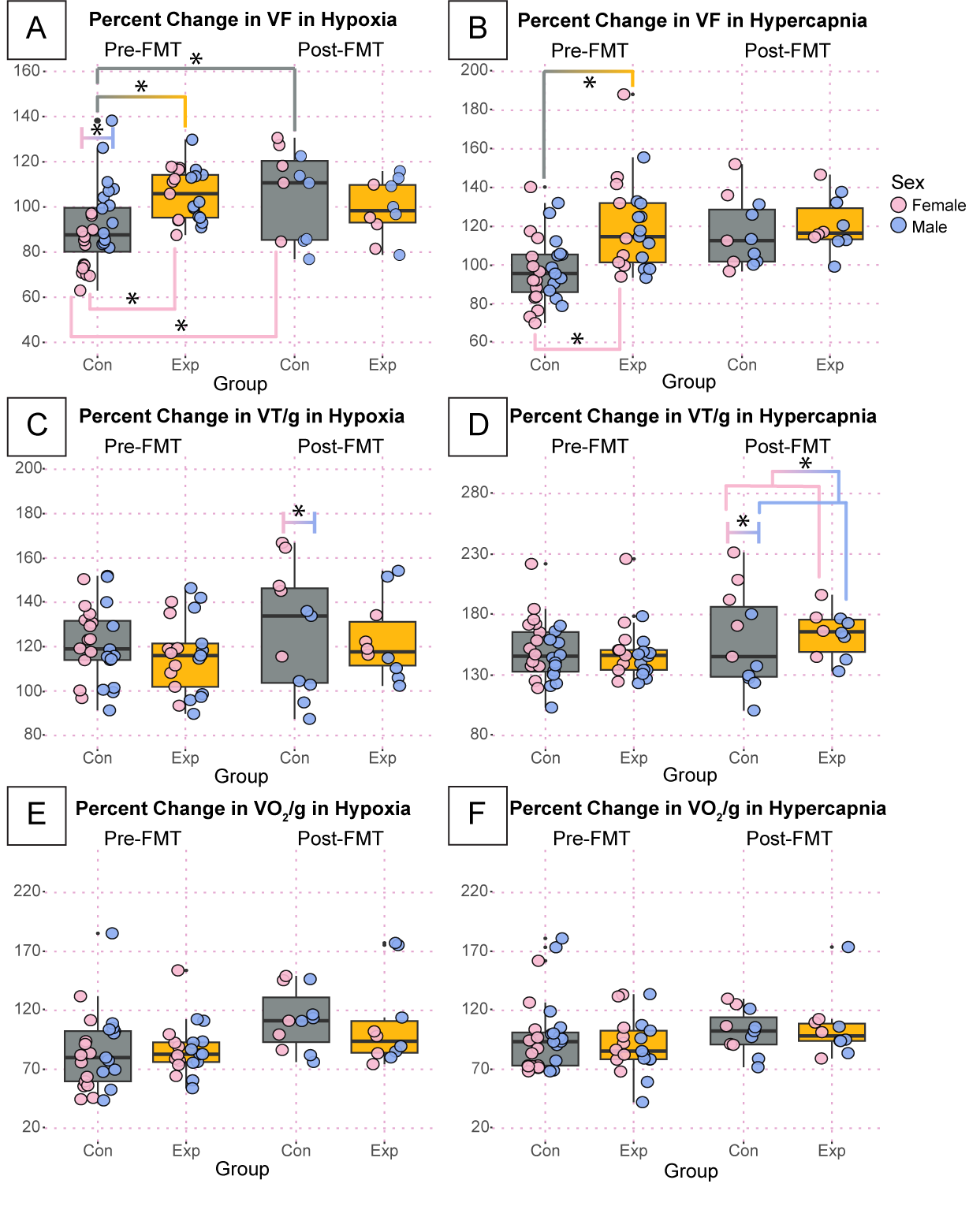

Supplement: Supplementary file 1 [file Image1.tif]
